# A frontal air intake may improve the natural ventilation in urban buses

**DOI:** 10.1038/s41598-022-25868-x

**Published:** 2022-12-08

**Authors:** F. Alexei Pichardo-Orta, Oscar Adrián Patiño Luna, J. Rodrigo Vélez Cordero

**Affiliations:** 1 Instituto de Física, Universidad Autónoma de San Luis Potosí, Álvaro Obregón 64, 78000 San Luis Potosí, S.L.P. México; 2 Investigadores por México-Instituto de Física, Universidad Autónoma de San Luis Potosí, Álvaro Obregón 64, 78000 San Luis Potosí, S.L.P. México

**Keywords:** Fluid dynamics, Engineering, Mathematics and computing, Physics

## Abstract

In this report we analyze the air flow across the open windows (natural ventilation) of an urban bus model and the consequent dispersion of aerosols emitted in the passengers area. The methods include computational fluid dynamics simulations and three ways to characterize the dispersion of passive tracers: a continuous concentration-based model, a discrete random model and a parametric scalar based on the so-called mean age of air. We also conducted experiments using a 1:10 scale bus model and $\text{CO}_{2}$ as a passive tracer to assess the ventilation characteristics. We found that dispersion and expulsion of aerosols is driven by a negative pressure in the standard bus design equipped with lateral windows. Also, the average age of air is 6 minutes while the air flow promotes aerosol accumulation to the front (driver’s area). To speed up the expulsion of aerosols and reduce their in-cabin accumulation, we propose a bus bodywork prototype having a frontal air intake. All the numerical models and experiments conducted in this work agreed that the expulsion of aerosols in this novel configuration is significantly increased while the average age of air is reduced to 50 seconds. The average air flow also changes with the presence of frontal air intakes and, as a consequence, the expulsion of aerosols is now driven by a frontal velocity field.

## Introduction

Covid-19 pandemic has motivated different research groups around the world to intensify the investigation of airborne contaminants. Although making definite conclusions have yet to wait more rigorous and controlled experiments^1,2^, an increased amount of evidence and study cases have stressed out the importance that has the air flow in the transmission and residence times of virion-containing droplets during the propagation of the pandemic^3–6^, particularly in places with little security such as confined, crowded or poorly ventilated areas^1,7^ (for transmission cases in the public transport see Refs.^3,8^, also see Ref.^9^ for an aerosol transmission experiment using animal models). A general call out has therefore been given to move or prefer activities outdoors, if possible^10,11^, together with regular practice of other security measures such as frequent ventilation^12^, mask wearing or the practice of physical distancing. The goal of this report is to offer general guidelines and propose novel designs to improve the ventilation and expulsion rate of aerosols emitted inside urban buses; in particular, these guidelines can help in emergencies as the one we recently experienced.

Studies on turbulent flows inside urban buses, together with the propagation of airborne species induced by such flows, have already been conducted by several research groups using computational fluid dynamics simulations (CFD). In a first group of papers we can find those that consider turbulent flows inside a bus generated by an air conditioning system^8,13–15^. In this situation, the position of air entries and exhausts, together with the amount of recirculated air or amount of fresh air added to the mixture, can be considered as design variables to test different ventilation modes and improve the expulsion of internal emitted aerosols. On the other hand, a second set of studies have simulated in-cabin turbulent flows formed at the expenses of the exterior air flowing through the open windows of the bus, or having no air conditioning system, as happens in many Latin American, Asian and African countries; these papers^8,16–19^ conform the main background of the present report. First of all, the majority of these studies have revealed a characteristic flow inside urban buses that may not be obvious at first glance: contrary to what we may expect, when a bus is moving at a certain speed having a fixed number of open windows, the exterior air enters from the back windows and then travels towards the front pushing or sweeping the aerosols from back to front, on average. This counterintuitive flow happens because pressure is lower at the front windows compared to the values at the back, causing this pressure driven flow. Another important observation taken from these studies is the fact that, not surprisingly, removal of indoor particles is accelerated when the bus windows are open^8^. In particular, Li and coworkers^16^ studied the flow characteristics and drag of contaminants generated by different arrangements of open windows; importantly, they remarked that opening the driver’s window together with the windows located at the middle of the bus can led to an observable “pumping effect” which transports air form back to front, as already mentioned. Li’s papers as well as other works^16,20,21^ have focused mainly in the transport of pollutants generated outside the bus, such as engine exhaust gases, which can then infiltrate towards the interior of the bus, or in the temperature distribution inside the bus generated by the flow field and its impact on thermal comfort levels^17,19^. Only Zhang *et al.*^8^ have explicitly considered the problem of aerosols emitted and transported inside a bus but without following their expulsion to the exterior (also consult the work of Mesgarpour^22^ on the spread of droplets inside a closed bus). Hence, it is necessary to conduct a new study on the transport and expulsion of aerosols occurring across the open windows of an urban bus by solving the external and internal flows simultaneously.

It is important to mention that ventilation quality or removal of pollutants from indoor spaces have also been studied computationally in other circumstances; relevant examples include the flow characteristics and motion of airborne species inside an aircraft cabin^23,24^, the flow inside a high speed train cabin^25^ and inside an automobile^26^ (these authors also noticed the aforementioned back-to-front pumping effect); flow and aerosol dispersion inside a conference room^27^, inside a generic ventilated room^28^, a supermarket^29^ or a restaurant^30^, and the flow inside buildings and hospitals (see Ref.^31^ for a general review on the subject). CFD simulations have also been usefull to asses the air quality and associated thermal comfort levels inside cars^32^, natural ventilated rooms^33^ and buildings within urban communities^34^, to mention a few.

## Outline and work hypothesis

**Figure 1:**
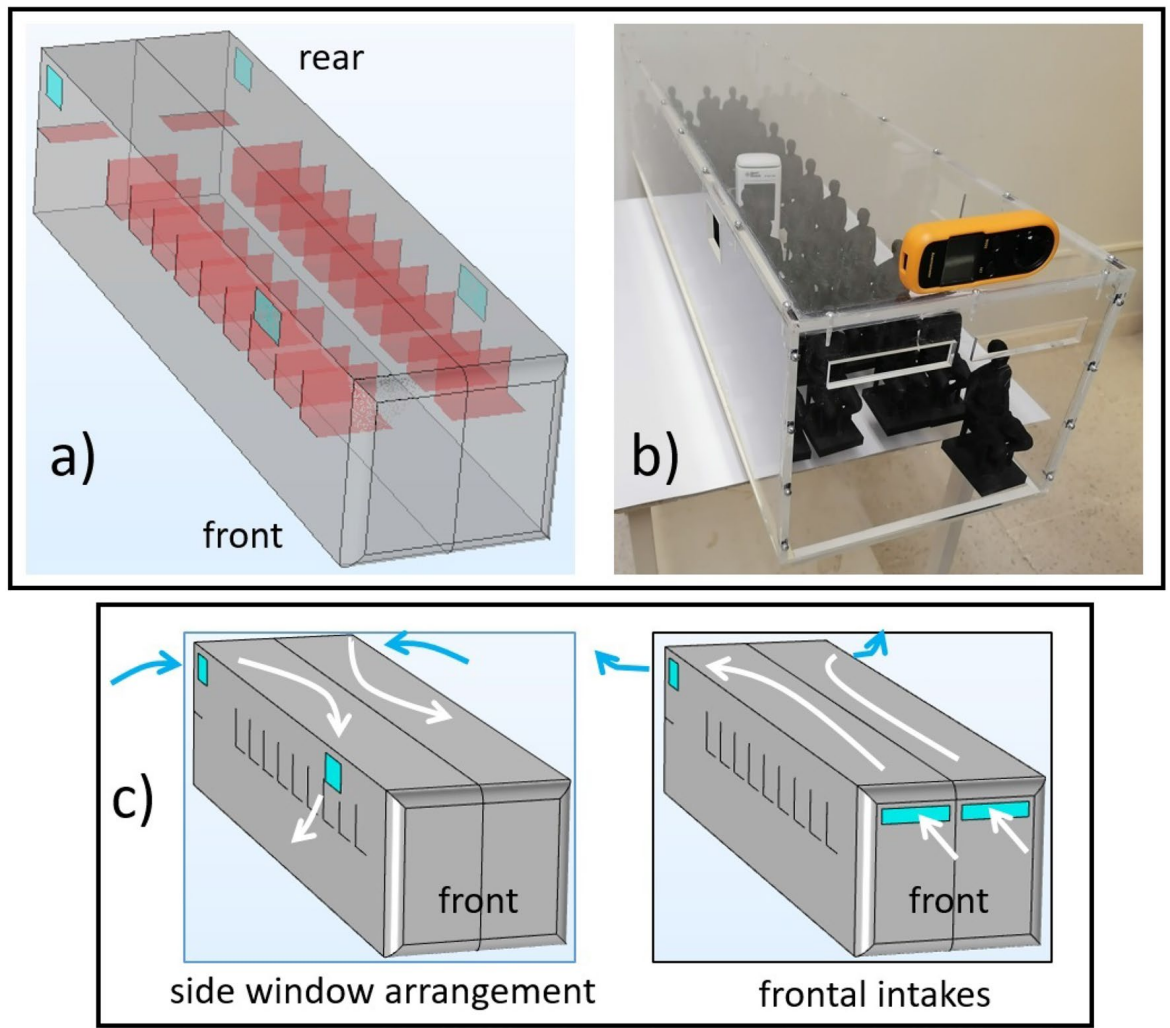
Image of the numerical (**a**) and experimental (**b**) models used in this study (for more details see the Methods section). Figure (**c**) shows a scheme of the mean air flow direction observed in the lateral windows configuration in which air is pumped from back to front, as well as the proposed configuration in which air inlets are installed at the frontal wall, changing the mean flow direction. In the numerical model (**a**) seats are denoted with red color while open windows are marked in blue. Numerical and experimental models were tested with and without passengers (manikins).

Figures 1a and b show the numerical and experimental models used in this report.

Briefly (the details can be consulted in the Methods section), the numerical model solves the internal and external air flow formed simultaneously along a real-sized bus traveling at a representative speed of $U=50 km/h$ (13.9*m*/*s*) and having a certain distribution of open windows. An example of what happens at lower speeds is presented in the supplementary material S1. Once the turbulent flow was obtained we proceeded to evaluate the ventilation of internal emitted aerosols using three different approaches. In a first approach we analyze the drag and expulsion of a continuous “cloud” of particles (concentration field) emitted by a Gaussian pulse having a width of 0.5 seconds and located at the center of the passenger’s area. To get a more realistic simulation of a breathing (exhalation) event, we also modeled the aerosol as a statistical ensemble of Lagrangian particles using a set of random parameters (particle velocity) in order to account for the stochastic nature of the aerosol release. Finally, since the ventilation characteristics of these Eulerian and Lagrangian representations depend on the site of the emission, we also computed the so-called mean age of air for a given flow field to visualize the re-change of indoor air. By definition, this parameter reports how difficult it is for a given emission to exit a control volume for any possible location of the emitter.

To be able to assess experimentally the ventilation characteristics inside the bus and explore new air intake locations; i.e., explore a novel distribution of open windows, we used an acrylic 1:10 scale bus model having or not 3D-printed passenger figures (empty or full bus) as our experimental setup (Fig. 1b). As the scaling parameter we chose to preserve the time it takes the air to travel the full bus length, i.e., $t=L/U$, where *L* is the length of a real bus ($\sim 10m$) or the length of our scale model (1*m*), while *U* is the traveling speed of a real bus (13.9*m*/*s*) or the speed of our scale bus (1.3*m*/*s*). Notice that this scaling option will reduce the Reynolds number by a factor of 100 but will, nevertheless, maintain the turbulent regime (from $2\times 10^{6}$ to $2\times 10^{4}$ in the experimental model). The Reynolds number is defined here using *U* and the width of the bus as the characteristic length. Additionally, we used a pulse of $\text{CO}_{2}$ and a commercial sensor to evaluate the ventilation characteristics in the experimental model given a certain arrangement of open windows.

Now we want to comment on the main hypothesis and motivation of this report, both which are illustrated in Fig. 1c. In current commercial bus designs, the windows are placed along the lateral walls of the bodywork, typically 4 to 5 sliding windows on each side. As mentioned in the introduction, this lateral arrangement promotes inner ventilation or particle sweeping from back to front, on average, due to the lower pressure field established at the wall of attack. The new configuration we want to explore is depicted on the right image of Fig. 1c and consists in collocating two open windows or air inlets at the front wall while leaving two lateral windows open at the back. We hypothesize that this configuration will not only change the average ventilation direction (front to back) but will also increase the expulsion of internal emitted aerosols. Although these new air inlets do not exist in commercial vehicles, it is worth saying that the current bus designs have available space in the area between the driver’s windshield and the bus roof (usually used to announce the bus route). It is this space where we put the two frontal windows in both, the computational and experimental models.

## Results

### 2D simulations: a first exploration

**Figure 2:**
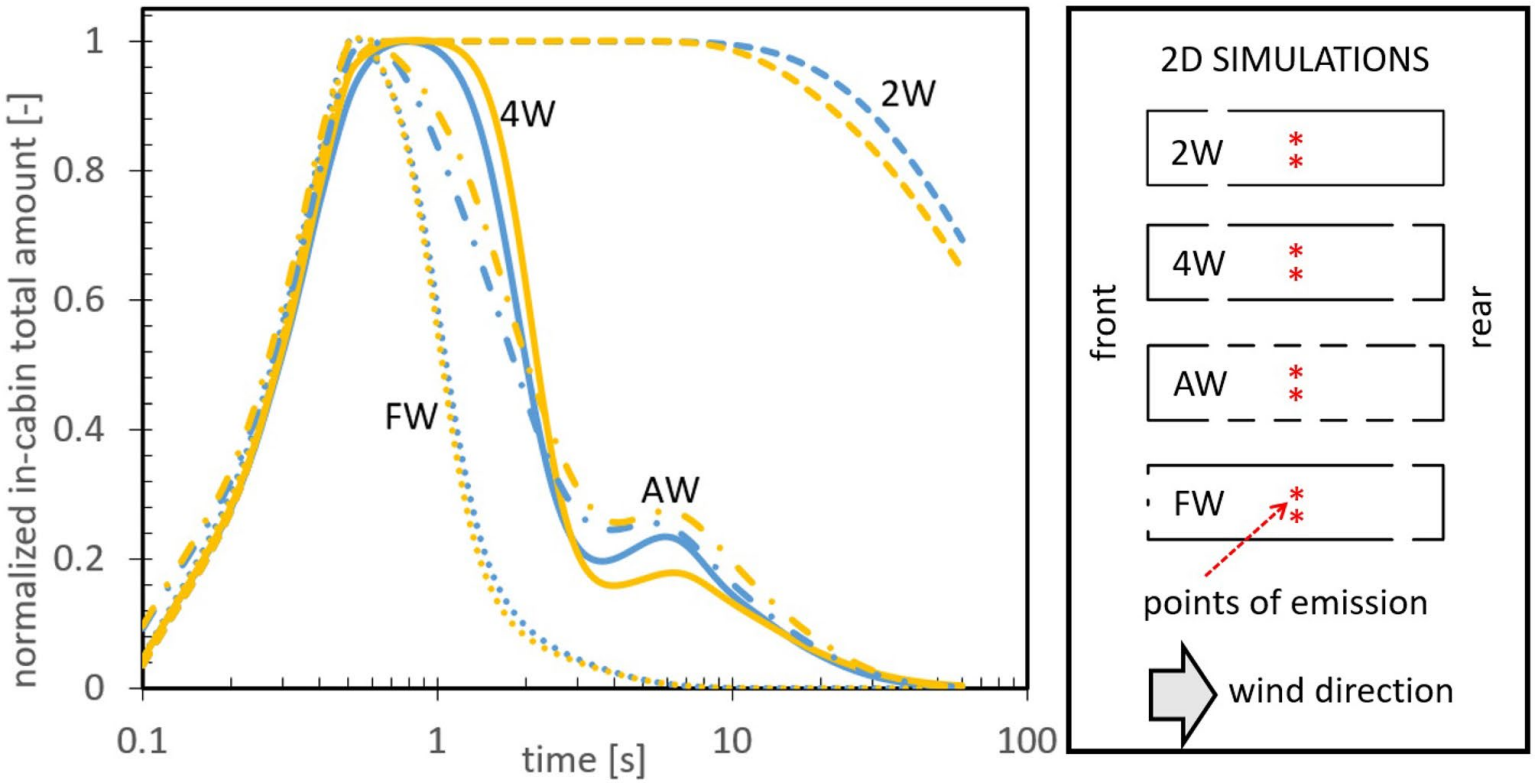
2D simulations: total amount of aerosols inside the bus as a function of time, normalized with the corresponding maximum values. Blue lines are the results using the $\kappa -\varepsilon$ model while yellow lines correspond to the SST $\kappa -\omega$ model. The explanation of the different open windows configurations are explained in the text and in the scheme at the right side. Aerosol emission occurs in the first 0.5 seconds at the center of the bus cabin. See also the movies included in the supplementary material. The local increments observed around the 6s in the AW and 4W cases indicate re-entry of aerosols promoted by the circulating currents formed outside the bus; i.e. part of the aerosols that left the bus at the frontal-lateral windows can return through the back-lateral windows.

We started by exploring the effects of the different open windows configurations by conducting 2D simulations on a horizontal plane coincident with the plane in which the windows are located. These simulations also helped us to optimize the numerical grid size and compare the $\kappa -\varepsilon$ turbulent model with the more rigorous, but demanding, SST $\kappa -\omega$ model (the details can be consulted in the Methods section and in the supplementary materials S2, S3 and S4, including validation tests for the SST $\kappa -\omega$ model, a grid-size dependency study and a Reynolds number dependency analysis). The different distributions of open windows that we studied are depicted in Fig. 2 and have the following nomenclature: 2W for 2 open windows, 4W for 4 open windows, AW for all windows open, and FW for the new proposed arrangement consisting in opening two air intakes at the front wall together with 2 lateral windows at the back. Additionally, in these 2D simulations we used the concentration-based model for aerosols, choosing the source of emission to be at the middle of the bus (marked with asterisks in the figure). The plot shows the total amount of in-cabin aerosols as a function of time, normalized with the corresponding maximum value or peak value, after the 0.5s emission pulse (the Gaussian tail starts at time $t=0$). Blue lines are the results using the $\kappa -\varepsilon$ model while the yellow lines correspond to the SST $\kappa -\omega$ model.

At first glance we can see that the rate of expulsion of aerosols increases as we go from 2 to 4 open windows, as expected (we have included some movies in the supplement so that the reader can have a clear picture of the dispersion characteristics). The 2W distribution permits the expulsion of aerosols even though it does not promote the establishment of the back-to-front pumping effect or air circulation since the back windows are closed. In this case, the observed lag time to start expulsion, $\sim 10s$, is due to the time that takes the air flow to reach the location of the open windows once released. The 4W and AW configurations show, on the other hand, a notable improvement of the expulsion rate because they increase the inner currents due to the establishment of the pumping effect (in the supplementary material S4 we have included some fluid flow maps to show this pumping effect). Therefore, it is very important for a proper circulation and renewal of air to leave at least two open windows at each lateral wall, two at the front and two at the back. As a brief comment, notice that the 2D simulations suggest that opening all the windows does not make a notable difference with respect to the 4W configuration, or just opening 2 windows at each side. Finally, we can see that the FW configuration achieved the best performance in terms of the expulsion rate; this gave us confidence to continue with the 3D simulations and pilot tests.

### 3D simulations: flow field and age of air

**Figure 3:**
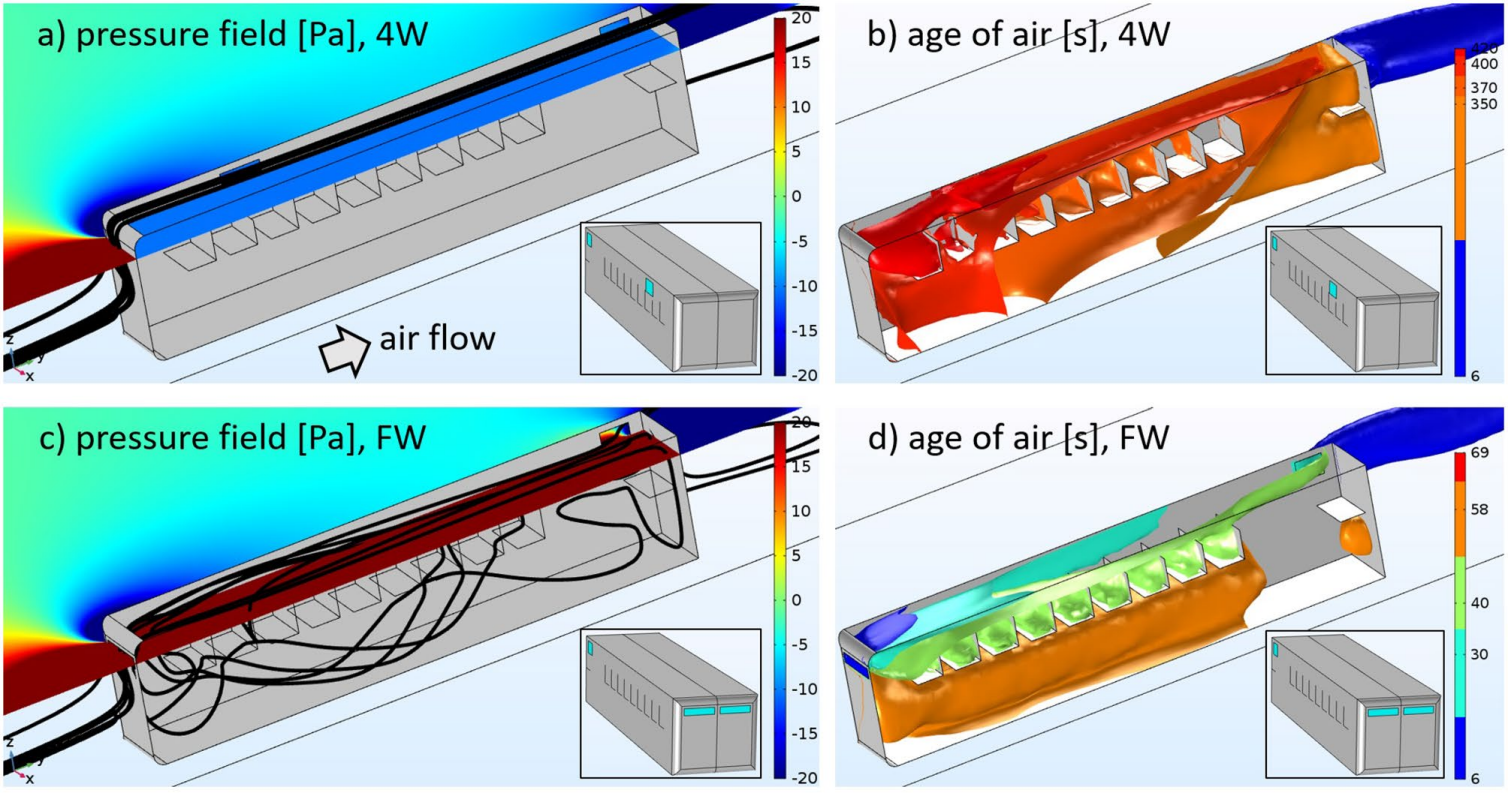
3D simulations: pressure field, streamlines (black lines) and mean age of air for the 4W and FW configurations (see also the insets as a guide). Air flow direction is indicated with an arrow; the roof of the bus is not shown to facilitate the view inside.

In the 3D simulations we analyzed in greater detail the lateral 4W and frontal FW configurations. Figure 3 shows some 3D-streamlines (black lines) together with a representative pressure field map, as well as the mean age of air for these two open windows distributions in an unoccupied bus. From Fig. 3a and c it is clear that while the pressure field at the windows level is similar in both situations outside the bus, the pressure sign in the cabin changes from negative (suction effect) in the 4W case, to positive (thrust effect) in the FW arrangement. The air streamlines also have a different destiny in each case: in the 4W configuration the streamlines coming from the front circumvent the bus bodywork without entering the cabin; in contrast, in the FW case they enter the bus through the frontal inlets, traveling the bus interior and exiting through the back windows.

The corresponding 3D-contour plots of the mean age of air are shown in Fig. 3b and d (a validation plot of the mean age of air, including its values inside and outside the bus cabin, is shown in the supplementary material S5). The age of air in the 4W case has an average value of 378s ($\sim 6$ minutes) with a maximum of 7.8 minutes located mostly at the frontal zones of the bus. For the FW configuration the mean age of air is 50s with a maximum of 69s. Among all the numerical results, this is perhaps the most remarkable difference between the negative pressure-driven flow found in the 4W case and the proposed FW configuration having the thrust effect. In addition, notice that since the age of air is complementary to the residence time values, i.e., the period of time a passenger spends inside the bus, for a fixed residence time it will be always safer to travel in the FW configuration than in the standard 4W in terms of aerosol accumulation.

### Statistical ensemble of aerosols

To obtain a more realistic simulation of the dissemination and expulsion of aerosols emitted inside the bus, in this section we show the results of tracking a statistical ensemble of discrete particles transported by the background flow, using random parameters to capture the stochasticity of an exhalation event (please consult the methods for a more detailed explanation of the release protocol). The exhalation event consists in releasing 100 particles during a period of 2 s and then follow their trajectories as a function of time; this event is replicated 40 times changing the release parameters randomly to have a total ensemble population of 4000 particles. The release site was the same as that chosen in the concentration-based model.

**Figure 4:**
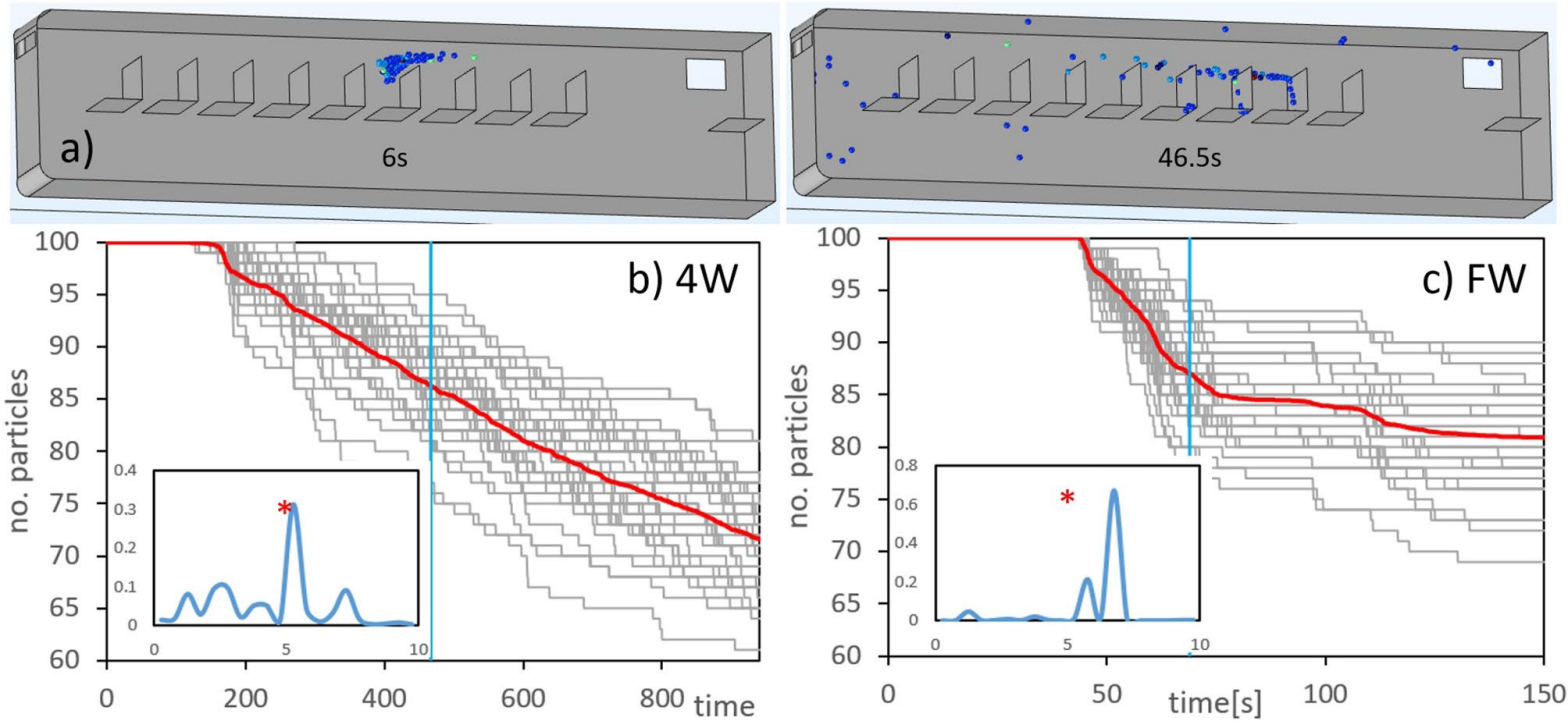
3D particle tracking; (**a**) example of the location of 100 particles 6 and 46.5 s after emission; (**b**) and (**c**): number of particles inside the bus as a function of time (seconds) for every exhalation event (gray lines) and for the ensemble average (red line). The vertical blue line in the main plots indicates the maximum age of air values obtained for the 4W and FW cases; the insets show the pdf(y) of the particle position at the last time of the simulations, the asterisks indicate the position of the particles release.

An example of an exhalation event is shown in Fig. 4a where we can see the location of the particle packet at 6s after emission, exiting at seat number 6, and then the same particles but now at time 46.5 s (see how some particles start leaving the bus through the rear window). Figures 4b and c show the number of particles inside the bus as a function of time for the 4W and FW configurations; the gray lines represent each replica of the ensemble while the red line is the ensemble average. We have also included in the insets the pdf(y) (probability density function) of finding the particles at the last time of the simulations in terms of the longitudinal distance (release site is at y=5m or seat $\#6$). It is important to say that we imposed the sticky condition to the particles ($u_{p}=0$ at the surface) so that they become immobilized whenever they touch a solid surface. This is a crude simplification because in reality, the adhesive forces of viral particles change according to the type of surface^35^ and depend on the wall’s law used in the transition and sub-viscous flow layers^36^.

**Figure 5:**
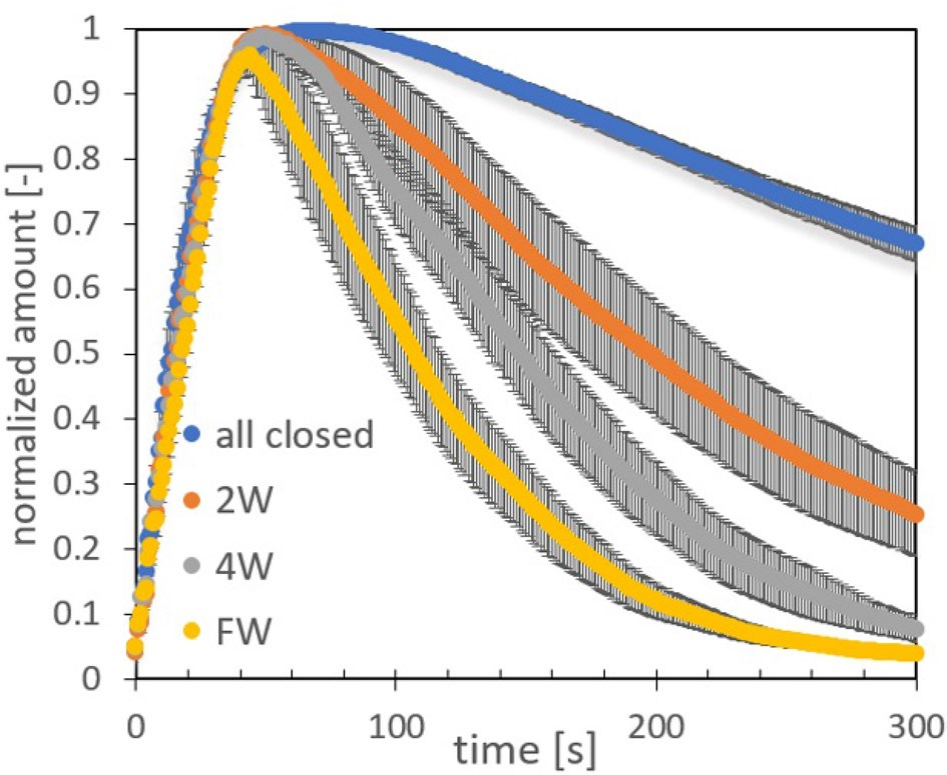
Ventilation experiments using $\text{CO}_{2}$ as the passive tracer in the empty bus. $\text{CO}_{2}$ concentration is normalized with the maximum value at the emission peak and measurements were done at the middle part of the bus. Error bars are indicated for 5 repetitions done for each condition.

**Figure 6:**
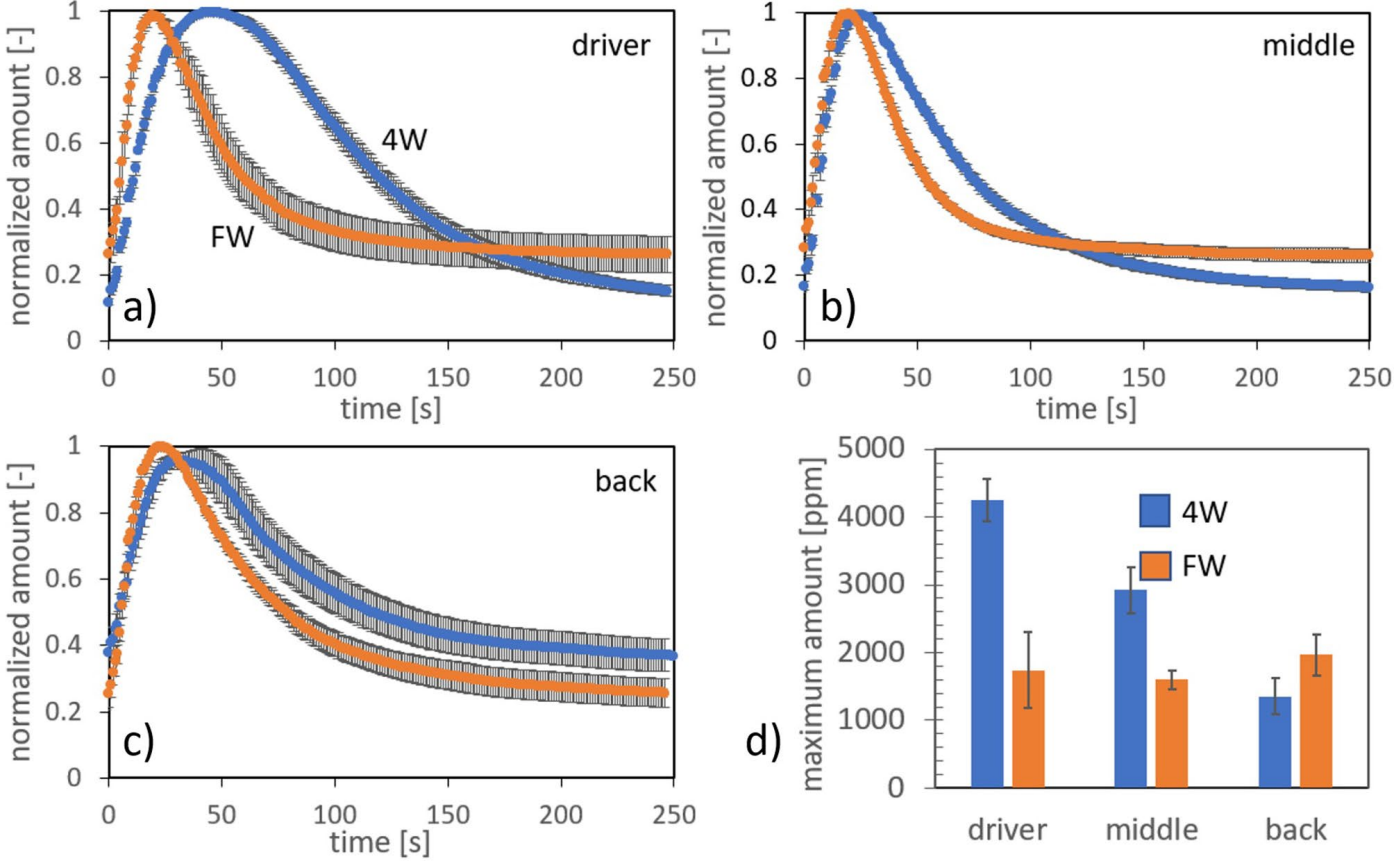
Ventilation experiments using $\text{CO}_{2}$ as the passive tracer in a occupied bus. (**a**)–(**c**): $\text{CO}_{2}$ concentration normalized with the maximum value at the emission peak as a function of time measured at the front (driver’s area), middle and back areas of the bus, respectively, for the 4W (blue line) and FW (orange line) configurations; (**d**): absolute $\text{CO}_{2}$ concentration detected at the emission peak for each case. Error bars are indicated for 5 repetitions done for each condition.

In the 4W case (Fig. 4b) we can see that after a retention time, the particles start exiting the cabin through the open windows at an average rate of 2 particles/minute. On the other hand, the pdf(y) shows a higher probability to find the particles near the release site and at the front. Particle expulsion is different in the FW case (Fig. 4c) because the retention time is followed by a rapid increase of the expulsion rate, $\sim 28$ particles/minute, but then decreases and reaches a stationary value. The corresponding pdf(y) shows that the localization probability is more narrow compared to the 4W case and is laden towards the rear of the bus. It is important to remark that the plateau observed in the corresponding main plot is due to the fact that most of the particles that have not left the bus at early times, become arrested at the solid inner walls. In the same sense, the continuous decrease observed in the 4W case reveals that the majority of the particles stay suspended in air. In summary, all this data indicate that the aerosol expulsion in the lateral 4W case is slow, continuous and maintain aerosols suspended in air for longer times than the FW configuration where the expulsion is faster, reach a steady state and promotes particle settling.

### Experimental essays

In this section we present the experimental results of the dispersion of $\text{CO}_{2}$ considering an empty bus and a bus full of passengers (see also Figs. 1b and 8a, b). As described in detail in the Methods, for the experiments we used a 0.5s pulse of $\text{CO}_{2}$ released at the middle of the bus model and then register the concentration evolution at different sites inside the bus. Figure 5 shows the time evolution of $\text{CO}_{2}$ released inside an empty bus for the following configurations: all windows closed, (2W) two open lateral windows, (4W) four open lateral windows and FW which is the frontal-lateral configuration. As we can see in Fig. 5, the dissipation rate increases as expected, obtaining the lowest expulsion rate when all windows are closed ($\text{CO}_{2}$ slowly escape through unsealed parts of the bus walls), and the largest one for the FW configuration. Figures 6a–c show the normalized $\text{CO}_{2}$ amount specifically for the 4W and FW cases at three different positions and having the bus full of passengers. The rate of decay or expulsion of $\text{CO}_{2}$ is, in all cases, higher in the FW configuration but the difference is particularly notorious in the driver’s zone. Figure 6d shows the maximum absolute value (ppm) of $\text{CO}_{2}$ detected after release for the three positions and the two configurations considered. Standout the fact that the maximum amounts are obtained in the position of the driver and middle part of the bus for the 4W case, although in the rear, the maximum amount is for the FW configuration.

## Discussion

The present study was inspired on an emergency situation in which airborne hazard particles, or aerosols, are emitted inside an urban bus and have to be expelled in the shortest possible time (maximum expulsion rates) to reduce their in-cabin accumulation. In the context of the emergency brought by COVID-19, the study of ventilation efficacy in the public transportation is highly pertinent since studies indicate^37,38^ that the highest risks of infection are found in urban buses compared to other modes of transportation.

The key message and proposal of this report is that the installation of a frontal casement window in the bus bodywork can substantially improve the ventilation quality in comparison with current designs in which air inlets are exclusively distributed along the lateral walls of the bus. We have shown with experiments and numerical simulations that the frontal FW configuration proposed here increases the expulsion rate of aerosols and reduces the maximum amount of suspended particles after release; it also reduces the particle spreading inside the bus and the mean age of air by one order of magnitude compared with the standard lateral windows configuration. As an additional note, the total flow rate in the FW configuration with the bus moving at 50 km/h and having an occupation of 50 people, yields a value of $\sim 100 L/s/person$, a value that is well above the recommended ventilation rate (8-10 L/s/person) proposed by the Scientific Advisory Group of Emergencies (SAGE) in UK^39^ during the COVID-19 emergency. This recommended rate will be accomplished by the FW configuration even if the bus moves at 9km/h.

In the analysis we have left aside other variables that are important in the design of an urban bus such as the total bus hydrodynamic drag, bus model specificity or other considerations such as the passengers comfort. In any case, our observations are useful to designers or engineers as it proposes a practical solution to an emergency situation in which the expulsion of aerosols becomes the relevant factor and where natural ventilation is the unique ventilation mode available, as it happens in middle and low-income countries. On the other hand, while this study focus on the ventilation characteristics of the basic bus’ bodywork, further studies should be done to investigate possible effects of the temperature differences between the outer regions of the bus (different environmental conditions) and the inner temperature (passengers heat release) as well as the subsequent thermal plumes caused by these temperature differences^40^. As a note, the average inner velocity in the FW case was 0.8 m/s, which is four times larger than the average velocity cause by the thermal plume as measured in stagnant conditions and using a manikin (0.24 m/s having $19.5^{\circ }C$ in ambient air^41^); it is however of the same order of magnitude as the average velocity found in the 4W configuration so it may impact the flow field in that case if temperature differences are large. As a parallel note, there is a recent work^42^ that reports the thermal plumes velocity measured above real people and found that the associated velocity is, on average, smaller (0.07 m/s) than the 0.24 m/s value previously measured using manikins. Therefore, we think that the issue of the temperature differences and their impact on the inner flows requires a whole new dedicated study considering temperatures below and above 30$^\circ$C or ambient conditions representative for countries located between the tropics.

We explored as well other open windows configurations that could, in principle, be attractive choices and improve the indoor ventilation. For instance, we constructed a geometry in which we modelled a driver’s window (see the supplementary material S6). This window opens rotating a hinge so that it can be oriented in a position of “attack” (windward) with respect to the outer flow. Surprisingly, we did not observe a notable improvement in terms of the back-to-front pumping effect observed when two sets of windows pairs are open. Urban buses have also mobile gates at the roof, but they in principle do not offer a notable improvement or changes to the mean velocity structure compared to the traditional lateral windows (see Ref.^18^ for more discussion on this regard).

**Figure 7:**
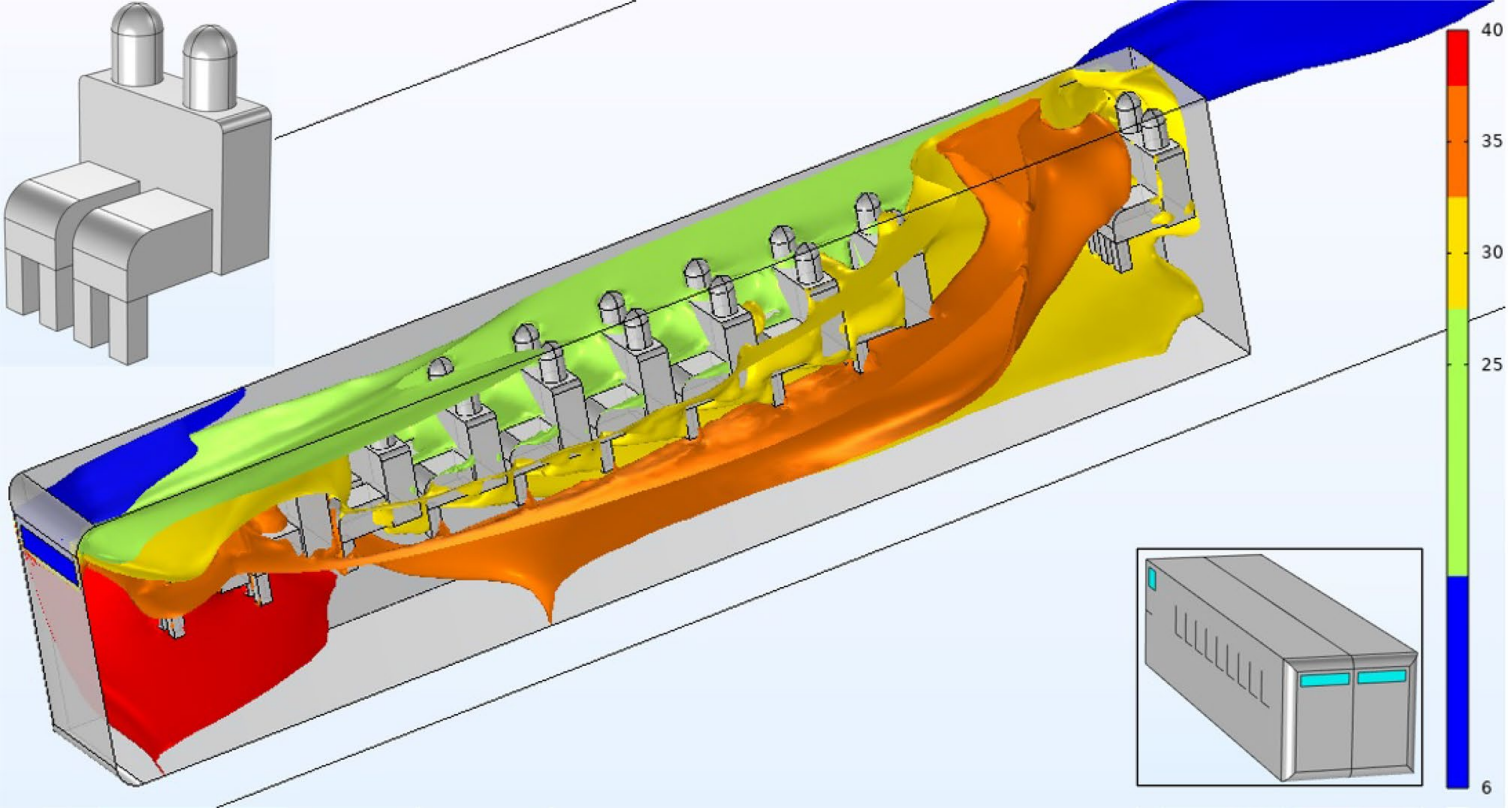
Age of air [s] contours obtained for the FW configuration including manikins. The upper inset shows the details of the manikin’s figure employed.

Another interesting question worth exploring is the effects that the mere presence of passengers can have on airflow or aerosol removal (besides the obvious fact that aerosols concentration increases with the number of passengers). To assess these effects in the proposed FW configuration, we repeated the corresponding simulations but now accounting for the presence of passengers (manikins) and applying a no-slip condition to the corresponding new surfaces. The results for the age of air distribution, ${\mathscr {A}}$, are presented in Fig. 7. It turns out that the presence of passengers preserve the order of magnitude of ${\mathscr {A}}$, but at the same time induces a reduction of its average value: 32s (max. 49s) in the occupied bus in comparison to the 50s (max. 69s) observed in the empty bus. Unexpectedly, this means that the presence of passengers increases the expulsion rate or diminishes the residence time of air inside the bus. Since the presence of passengers reduces the volume occupied by air, these numerical results suggest that the reduction of the internal volume fraction of air has more impact on the increment of the expulsion rate than the hindering effect caused by the no-slip boundary condition applied to the surface of the passengers (hydrodynamic effect). The increment of the expulsion rate associated with the presence of passengers was corroborated by the experiments upon comparing the evolution of the $\text{CO}_{2}$ amount with and without manikins, see the supplementary material S7. In the supplementary material S8 we also included a second simulation using high-resolution manikins for the FW case, obtaining a similar trend as that shown in Fig. 7 (that simulation was not included in the main text because it didn’t reach a numerical tolerance below $10^{-3}$).

In connection with the turbulent models used in this work, we did not detect important differences between global expulsion rates of aerosols obtained with the $\kappa -\varepsilon$ or the SST $\kappa -\omega$ models, see Fig. 2. As shown in the supplementary material S4, the flow field does present some differences depending on the turbulent model used. We also included a comparison between the expulsion rates obtained with these two models but without including the turbulent mixing term in the diffusion-convection equation (see the methods section and S3). In that case, the difference in the expulsion rates is very notorious, highlighting the fact that one should always include turbulent mixing in the governing mass transport equations to obtain reliable results. In the supplementary material S4 we also included the results of a 2D simulation varying the Reynolds number by a factor of 100. It is shown that a change of Re within the turbulent regime preserves the structure of the average flow field but decreases, by the same factor, the turbulent parameters such as the turbulent kinematic viscosity. Therefore, it is expected that the experimental results presented here using the scale bus model will not exactly reflect a real scenario in the sense that the fluctuating part of the flow will be more intense in a real-sized bus and so will be the aerosol dispersion. This imposes a conceptual limitation to the experiments presented in this report.

Finally, it is our hope that the present report can contribute to the general understanding of the physics and natural flows occurring inside urban buses and help decision makers to opt for design alternatives that may improve indoor ventilation in an emergency or critical situation. The current numerical results can also be used as a starting point for more advanced RANS models such as the Reynolds stress transport model, complemented with the so-called two-layer model and which have shown to improve particle deposition predictions^36,43^, or for Large Eddy Simulations or Direct Numerical Simulations which can provide more information about the turbulent and transport fluctuations of the indoor emitted aerosols.

## Methods

### Numerical model and finite element specifications

**Figure 8:**
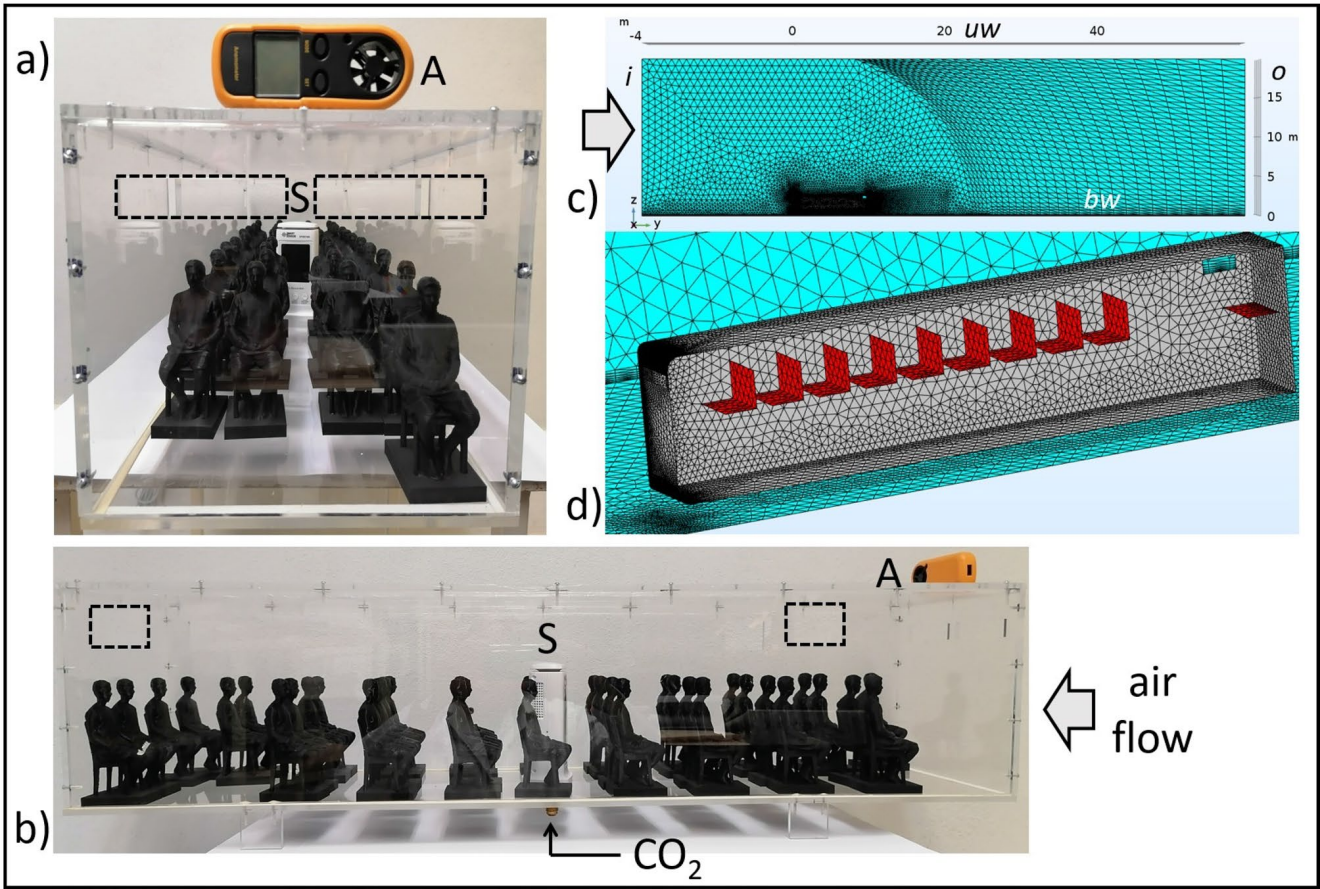
Numerical and experimental setups. (**a**) and (**b**) show the acrylic bus model (L = 1 m), including the 3D-printed passenger figures, the $\text{CO}_{2}$ feeding which is located at the middle of the bottom wall, the $\text{CO}_{2}$ sensor (S) and the anemometer (A). The position of the windows are marked with dashed lines to guide the eye. (**c**) shows a panoramic view of the whole numerical domain, including the inlet wall (i) where air enters the domain, the outlet wall (o) and the upper wall (uw) which are considered open boundaries, as well as the bottom wall (bw) on which the no-slip condition is applied. The denser mesh region is where the bus is located; (**d**) zoom of the numerical domain showing the bus bodywork (gray) and the external walls (blue); the seats are marked in red.

Figure 1 shows the numerical and experimental models used in this report. In the numerical model we generated a real sized bus having a length (L) = 9.92 m, width (W) = 2.5 m and height (H) = 2.2 m, leaving a gap of 0.42*m* between the internal floor of the bus and the ground level. Bus solid walls and internal seats were assumed to have zero thickness so that the bus bodywork is in fact a 2D numerical mesh embedded in a 3D numerical domain. The whole numerical bus was placed inside a 3D rectangular box of size $8L\times 2L\times 2L$, see Fig. 8c and d, placing the bus closer to the frontal external wall *i* (cartesian coordinates origin is placed at the frontal wall of the bus, with *y*-axis oriented with the bus length). The open windows are regions at the bus’ solid walls where the no-slip condition is not applied. The lateral windows have a size of $53\times 40$cm while the frontal windows have $90\times 20$cm each. A finer tetrahedral mesh was generated at the bus walls, interior, and zone immediately behind the bus’ rear wall where turbulent helical vortex structures are usually formed and which require higher spatial resolution^44–46^. A total of $1.3\times 10^{6}$ and $3\times 10^{6}$ elements were used in the 3D models for the unoccupied and occupied bus, and $5.9\times 10^{4}$ in the 2D models (all the simulations were assumed to have left/right symmetry, except the AW case, so the number of elements only reflects half of the whole domain).

The simulations were ran similar to a wind tunnel setup; i.e., the bus is fixed in space while the wind enters through the external wall “*i*” shown in Fig. 8c at $U=50\, \text{km}/\text{h}$. The rest of the external walls “uw” and “*o*” are assumed to be open boundaries (stress free condition), while at the bottom wall “bw” or ground level we applied the no-slip condition. When manikins were included in the model, the no-slip boundary condition was also applied to the corresponding surfaces. The external Reynolds number was $Re\approx 2\times 10^{6}$ based on the bus width. Simulations were ran in COMSOL Multiphysics; for more details on the solver configuration please consult the supplementary material S2.

### Fluid dynamics

Air flow is assumed to be isothermal, incompressible and has no changes in the presence of aerosols (dilute regime^13^). In this case, we can decouple the fluid flow from the transport equations and solve them in two sequential steps. We then solved first the average steady velocity ${\overline{u}}_{j}$ and average pressure ${\overline{p}}$ following RANS models:^47,48^

1 $$\begin{aligned}{} & {} \nabla \cdot {\overline{u}}_{j}=0 \end{aligned}$$

2 $$\begin{aligned}{} & {} \rho {\overline{u}}_{j}\frac{\partial {\overline{u}}_{i}}{\partial {x}_{j}}=-\frac{\partial {\overline{p}}}{\partial {x}_{i}}+\frac{\partial }{\partial {x}_{j}}\left[ (\mu +\mu _{T})\frac{\partial {\overline{u}}_{i}}{\partial {x}_{j}} \right] \end{aligned}$$

where $\rho$ and $\mu$ are the medium density and viscosity, respectively, while $\mu _{T}$ is the turbulent viscosity obtained either with the $\kappa -\varepsilon$ or the SST $\kappa -\omega$ turbulent models. Whenever we tried to find a convergent solution using SST $\kappa -\omega$, we started using $\kappa -\varepsilon$ to find a first solution. Standard wall functions were applied to $\kappa -\varepsilon$ on the walls having no-slip condition, while the Wolfshtein wall model^49^ was applied to SST $\kappa -\omega$. On the other hand, since both models need to solve the turbulent kinetic energy $\kappa$, we fed the model with values of $\kappa$ applied on the inlet wall “*i*” (see Fig. 8c) obtained from wind tunnel experiments^45^. In the supplementary material we have provided more references regarding previous validation tests done to the SST $\kappa -\omega$ model.

### Diffusion-convection equation

Once we solved for the average fluid flow in steady state, we then proceeded with the aerosol transport simulations using two different approaches. In the first one we considered a concentration-based approach (continuous formulation) of the aerosol cloud, where ${\overline{c}}$ represents the average concentration field and whose governing equation can be written as:

3 $$\begin{aligned} \frac{\partial {\overline{c}}}{\partial t}+{\overline{u}}_{j}\frac{\partial {\overline{c}}}{\partial x_{j}}=\frac{\partial }{\partial x_{j}}\left[ \Gamma _{c}\frac{\partial {\overline{c}}}{\partial x_{j}} \right] +S(x_{o},t)\;, \end{aligned}$$

where

4 $$\begin{aligned} \Gamma _{c}={\mathscr {D}}+{\mathscr {D}}_{T}={\mathscr {D}}+\frac{\mu _{T}(\vec {x})}{\rho \; Sc} \end{aligned}$$

being ${\mathscr {D}}$ the mass diffusion coefficient and ${\mathscr {D}}_{T}$ the turbulent diffusion or turbulent mixing. Additionally, *Sc* is the turbulent Schmidt number whose value is nearly a constant, $\sim 0.7$, for a wide range of Re numbers^50^, but can be alternatively defined in terms of the flow field variables^51^. To consider the emission of aerosols beginning at time $t=0$, we have included a source term in Eq. (3) which has the form $S(x_{o},t)={\dot{q}}\;pulse(t)\;{\hat{c}}|_{node:x_{o}}$. Here *pulse*(*t*) is a smooth pulse function with a standard deviation of 0.5s, ${\hat{c}}$ is the test function for the concentration field in the finite element formalism and ${\dot{q}}$ is the strength of the emission occurring at the node $x_{o}$. As a case of study, the point source is placed at $x_{o}=\{\pm 0.6,5,1.6+0.42[m]\}$. Since we are assuming that the aerosols are so diluted that they do not change the hydrodynamic properties of air, in practice ${\dot{q}}$ can have any value; therefore, we can present the evolution of ${\overline{c}}(x,t)$ normalized with the maximum value or peak of the pulse. Specifically, in Fig. 2 we showed the total amount of in-cabin aerosols, ${\mathscr {N}}(t)|_{in}$, as a function of time, normalized with the maximum value appearing at time $t_{max}$, i.e.,

5 $$\begin{aligned} {\mathscr {N}}(t)|_{in}=\frac{\int _{in}\;c(x,t)\;d{\mathscr {V}}}{\int _{in}\;c(x,t_{max})\;d{\mathscr {V}}} \end{aligned}$$

### Lagrangian formulation of aerosols

The aerosol cloud was also modelled explicitly by following a set of Lagrangian spherical particles emitted at the same site $x_{o}$ as in the continuous formulation. In this way, the expulsion of aerosol droplets is described explicitly and there is no need to use a pulse emission with an associated point strength. On the other hand, note that since the typical relaxation time of particles having sizes of order $\sim 1\mu \text{m}$ is around $9\times 10^{-9}s$, the characteristic Stokes number is small and the droplets should follow closely the background flow, similar to the continuous formulation. Newton’s equations were solved by considering the drag force $F_{d}$ as follows:

6 $$\begin{aligned} m_{p}\frac{d\;u_{p,i}}{d\;t}=\vec {F}_{d}=\frac{m_{p}}{C_{c}}\frac{3\mu C_{D} Re_{r}}{4\rho _{p}d_{p}^{2}}({\overline{u}}_{i}-u_{p,i}) \end{aligned}$$

where $m_{p}$, $\rho _{p}$, $d_{p}$ and $\vec {u}_{p}$ are the mass, density, diameter and velocity of the particles while ${\overline{u}}_{i}$ is the average steady velocity obtained with the RANS models. Here we are interested in following the path of aerosol particles emitted during normal breathing; therefore, we considered the particle distribution found experimentally in Ref.^52^ and whose particle size range is 0.7–5.5$\mu {m}$ with peak at 1.5$\mu {m}$ (we considered only probability densities higher than $10^{-2}$, smaller particle size fractions were neglected). For these particles sizes, buoyancy forces can be neglected ($\rho _{p}=1000kg/m^{3}$) as well as evaporation dynamics due to its small characteristic time scale^30^. On the other hand, with these particle sizes the Knudsen number is not so small, $Kn\sim 0.1$, so the Cunningham slip correction^53^ is applied to the drag force as:

7 $$\begin{aligned} C_{c}(Kn)=1+Kn\left[ 2.514+0.8\exp {\{-0.55/Kn\}} \right] \end{aligned}$$

although we did not detect significant changes when $C_{c}$ was not included. The drag factor $C_{D}(Re_{r})$ was calculated using standard tables valid up to $Re_{r}\sim 10^{6}$, although the results are similar if the Schiller-Naumann^54^ empirical formula is used instead. Here the relative Reynolds number is defined as $Re_{r}=\rho |\vec {u}-\vec {u}_{p}|d_{p}/\mu$. To simulate the exhalation event, we released 100 particles at $x_{o}$ for a time window of 2s having a uniform distribution of unit vectors coming out from a $47^{\circ }$ cone^55^ pointing towards the front wall of the bus. In order to account for the randomness of the aerosol distribution during an exhalation, we repeated the exhalation event 40 times, each time picking different delivery times for each particle within the 2s window and different speeds or velocity magnitudes using a random generator number. The exhalation speed range was 0 to 20m/s, being the upper limit the characteristic velocity of a cough^56^. As an additional note, taking into account the Brownian motion expressed in terms proportional to $\sim \sqrt{k_{B}T}$ did not make substantial difference in the simulations. Finally, the sticky condition was applied to the particles whenever they touch a no-slip surface, i.e., they become arrested ($u_{p}=0$).

### Mean age of air

While the common transport equations (Lagrangian or Eulerian) allow us to see explicitly the drag of aerosols and its subsequent expulsion through the open windows, their application is somehow limited because each chosen point of emission will experience a different hydrodynamic drag depending on its particular location; in order words, we will need to probe a considerable number of different point sources in order to have a complete map of the retention time (or expulsion rate) of the aerosols and detect regions with poor ventilation. Therefore, in order to get a more general picture of the ventilation quality inside the bus, we also computed the so-called mean age of air, ${\mathscr {A}}$^57^, in the outer and inner regions of the bus. The interpretation of ${\mathscr {A}}$ may be given in the following terms: imagine that the bus receives, at a certain time $t=0$, a bundle of particles moving with the incoming flow departing from wall “*i*” (Fig. 8c); then, at some location in or outside the bus, an observer detects the particles at the moment they pass through; the scalar field ${\mathscr {A}}$ will be then the average time that has passed since the release of the particles at the inlet wall until the observer detects such particles. Formally, the local value of ${\mathscr {A}}$ is defined as:

8 $$\begin{aligned} {\mathscr {A}}(x)=\frac{\int _{0}^{\infty }\;t\;c(x,t)\;dt}{\int _{0}^{\infty }\;c(x,t)\;dt}\;. \end{aligned}$$

As shown by Li and Tilton, Sandberg and Spalding^57–59^, the transport Eq. (3) can be manipulated in order to accommodate the definition given by (8) to obtain a partial differential equation for ${\mathscr {A}}$ in the form:

9 $$\begin{aligned} \nabla \cdot \left( {\overline{u}}_{i} {\mathscr {A}} \right) =\nabla \cdot \left[ \Gamma _{c}\;\nabla {\mathscr {A}} \right] +1\;. \end{aligned}$$

As commented by Liu and Tilton, Eq. 9 has the same form as the steady transport equation for diluted species with an extra term corresponding to a reaction term and equal to 1. In this sense, we can solve ${\mathscr {A}}$ using the mean velocities given by the turbulent models and using the same numerical solvers used for regular PDEs. In the supplementary material we have included a validation test for ${\mathscr {A}}$ which shows that its value at the outlet wall “o” (see Fig. 8c) is in good agreement with the total time that the air spends travelling the whole computational domain, i.e., $8L/U=79.3[m]/13.9[m/s]=5.7s$. Finally, for the field ${\mathscr {A}}$ we applied the Dirichlet condition ${\mathscr {A}}=0$ at the inlet “*i*”, while in the rest of the walls, including the solid walls and outlets, we applied the Neumann condition $\overrightarrow{n}\cdot \nabla {\mathscr {A}}=0$.

### Experimental essays

Experiments were performed using an acrylic 1:10 scale model of an urban bus having or not 3D-printed figures of seated people, see Figs. 1b, 8a and b. As the scaling parameter we opted to fix the time it takes the air flow to travel the full bus length, i.e., $\sim 0.7s$. An anemometer (A) was used to adjust the air speed, $\sim 1.3m/s$, as provided by a regular fan placed at the front of the model. We used $\text{CO}_{2}$ as a passive tracer to infer the ventilation characteristics given a certain distribution of open windows (the windows in the model are simply holes made on the acrylic walls and closed if necessary with acetate sheets). Pulses of $\text{CO}_{2}$ 0.5s long were introduced inside the bus through a connection placed in the middle part of the bus (see Fig. 8b). A wireless commercial $\text{CO}_{2}$ sensor (S) and recorder (ST8310A, 0-20,000 ppm) was placed at the middle part, back and front positions to follow the $\text{CO}_{2}$ concentration inside the model after release at time $t=0$. Five repetitions were done for each condition, detaching the upper cap of the model between consecutive measurements to dissipate the remaining $\text{CO}_{2}$. As a final comment, it is important to remark that although the release and detection of $\text{CO}_{2}$ is easy to implement experimentally and provide a measure of the ventilation characteristics inside the bus, discrepancies between the experimental and numerical models can appear due the different emissions implemented in each model: microscopic particles in one and a gas in the other.

## Supplementary Information

Supplementary Information 1.

Supplementary Information 2.

## Data Availability

All data generated or analysed during this study are included in this published article [and in the supplementary material].
