# Supplementary material for: A frontal air intake may improve the natural ventilation in urban buses
Source: Sci Rep. 2022 Dec 8;12:21256. doi: 10.1038/s41598-022-25868-x (PMC9732044; doi:10.1038/s41598-022-25868-x)
Supplement: Supplementary file 1 — Supplementary Information 1. [file 41598_2022_25868_MOESM1_ESM.pdf]

SUPPLEMENTARY MATERIAL

A frontal air intake may improve the natural ventilation in urban buses.

F. Alexei Pichardo-Orta[1], Oscar Adrián Patiño Luna[1] and Juan Rodrigo Vélez Cordero[2,*]

[1] Instituto de Física, Universidad Autónoma de San Luis Potosí, Álvaro Obregón 64, 78000 San Luis Potosí, S.L.P. México.
[2] Investigadores CONACyT por México – Instituto de Física, Universidad Autónoma de San Luis Potosí, Álvaro Obregón 64, 78000 San Luis Potosí, S.L.P. México.

Email[*]: jrvelez@ifisica.uaslp.mx

S1. Effect of the inlet velocity

Figure S1 shows the dissipation of the normalized in-cabin amount of aerosols for the FW configuration setting different air speeds applied on the inlet boundary *"i"* (see Fig. 8c). It can be observed that the expulsion rates (slopes of the curves) are similar to the original value found for 50km/h, although the time to start expulsion becomes longer as speed decreases due to the increased travel time it takes for the aerosol cloud to reach the windows. In quantitative terms, the time needed to decrease the original amount of aerosols to 0.1% obeys a power law scale, $301.6(speed)^{-1}$; in the 2D simulations; as an example, for 50km/h, the computed time is 6s, while for 10km/h is 30s.

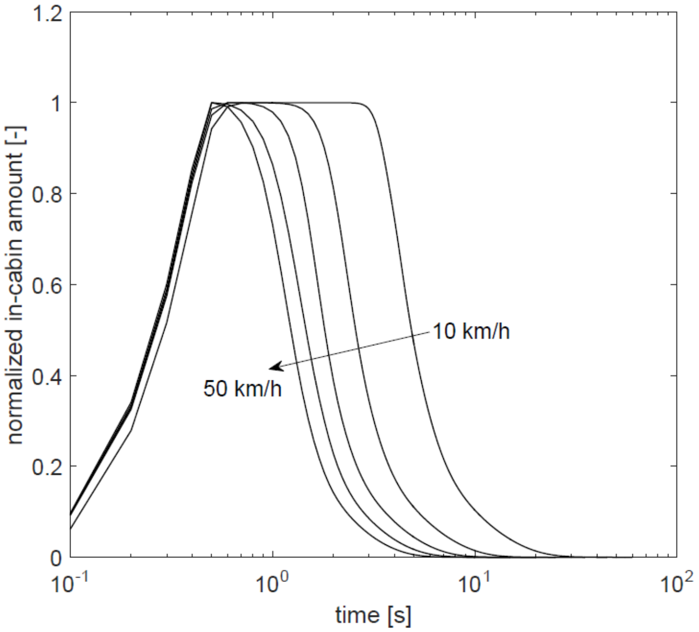

Figure S1. Dissipation of the in-cabin amount of aerosols in the FW configuration. The different speeds are 10, 20, 30, 40 and 50 km/h. Simulations were done in 2D using the SST $\kappa - \omega$ model.

S2. Mesh size dependency study and solver configuration

Figure S2 shows the dissipation of the total in-cabin amount of aerosols for the AW configuration (all windows open). In this mesh size dependency study, we varied the mesh defined at the walls of the bus to increase the spatial resolution of the in-cabin zone; the rest of the mesh far from the bus was unchanged. For the coarser mesh we used a size of 0.1W (being W=2.5m the bus width) at the lateral walls of the bus, while at the rear wall we used 51×10^{-3}W. For the finer mesh we used 6.4×10^{-3}W at the lateral walls and 3.2×10^{-3}W at the rear wall. The plot shows that the use of a very coarse mesh renders a dynamic profile clearly different from the rest of the curves (thicker line). In the current simulations we selected a mesh size corresponding to the dashed line (fine mesh).

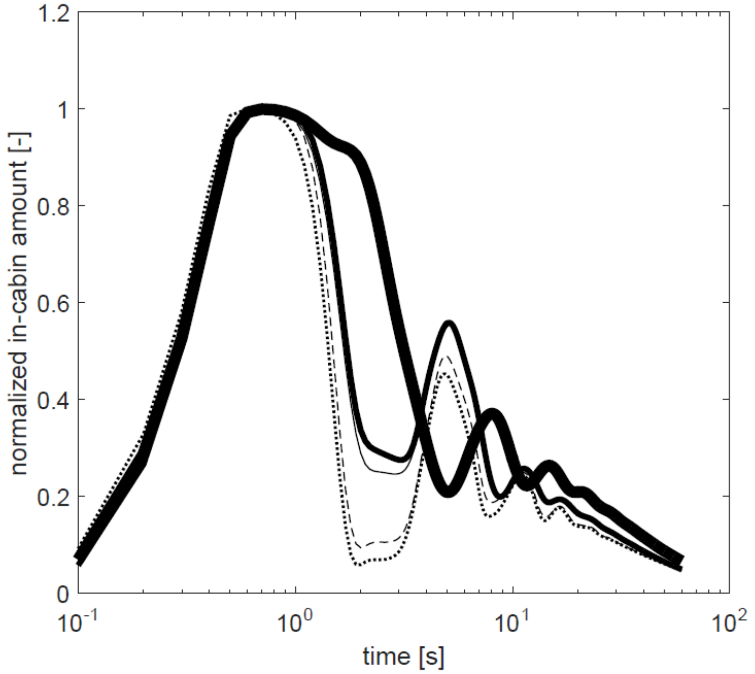

Figure S2. Mesh size dependence study using the AW configuration (all windows open, 2D); thicker, medium, and thinner solid lines correspond to a total number of elements of 1.6×10^4, 2.1×10^4 and 3.4×10^4, respectively. The fine (dashed line) and finer (dotted line) meshes correspond to a number of 5.9×10^4 and 11.9×10^4 elements, respectively.

In all the simulations we used a segregated solver, that is, the averaged Navier Stokes equations are solved in a different step from the computation of the turbulent variables within the algorithm loop. On the other hand, for 2D problems, the solver computes the unknown variables vector using matrix factorization (direct solvers such as LU decomposition and its variants). For 3D problems, an iterative solver is chosen so the unknown variables are solved approximately using the Generalized Minimum RESidual method and a preconditioner found by using the algebraic multigrid method (within this last step, the solver finds and initial guess using a coarser mesh and a direct solver). Maximum convergence tolerance was set to 10^{-3}.

S3. Simulations without turbulent mixing

In Figure 2 of the main text, we demonstrate that aerosol expulsion rates obtained with both turbulent models, the $\kappa - \epsilon$ and SST $\kappa - \omega$, are very similar. In Figure S3 we show the same simulations but without considering turbulent mixing in the diffusion-convection equation. It is remarkable that now the differences are accentuated; see for example the 2W case where the average flow obtained with $\kappa - \epsilon$ does not even predicts the expulsion of aerosols. This reveals that, although the mean fluid flow obtained with both models are not exactly the same (as shown next in S4), the fluctuating terms considered in the turbulent mixing smooth out such differences.

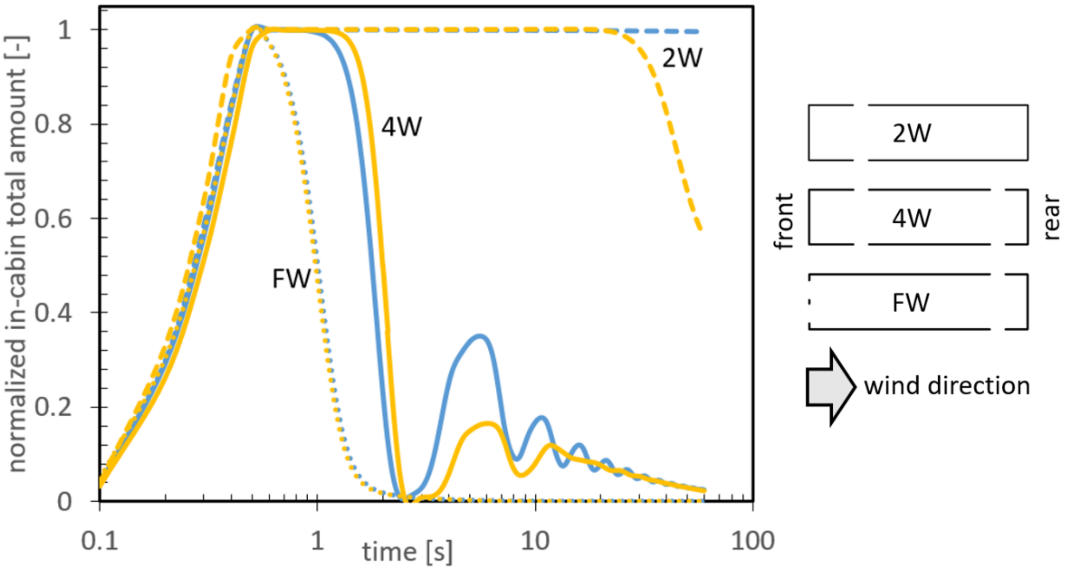

Figure S3. Normalized in-cabin amount of aerosols as a function of time for the 2W, 4W and FW configurations. These simulations were conducted without considering turbulent mixing in the diffusion-convection equation. Blue lines denote the results using $\kappa - \epsilon$, while yellow lines correspond to the SST $\kappa - \omega$ model. Compare this Figure with Figure 2 of the main text.

S4. 2D fluid flow maps and Reynolds number dependency analysis

The $\kappa - \epsilon$ turbulent model is extensively used due to its versatility and adaptability in different geometries; however, it is known that is not optimal for flows close to walls or past obstacles with strong vortex formation, detachment of streamlines or adverse pressure gradients [1, 2]. On the other hand, low or mixed Re formulations, like the SST $k - \omega$ model, have shown to improve turbulent flow predictions and have been validated against different experimental data such as flows past a cube in channel flows [3], flow around a hydrofoil at standard temperatures [4], flow across a train at different angles of attack [5], ventilation inside a cubical enclosure [6] and flow and deposition of aerosols in lung airways models [7], to mention a few (these papers also highlight similarities between SST $k - \omega$ and other formulations such as RNG $k - \epsilon$).

On the other hand, in this work we have also seen that either $\kappa - \epsilon$ or SST $\kappa - \omega$ yield similar results for the global expulsion rates. There are, however, some important quantitative differences that we now want to remark. To facilitate the analysis and visualization of the flow fields generated by both models, we have computed the so-called Okubo-Weiss parameter, $\mathbb{Q}$, in the 2D simulations defined as [8]:

$$\mathbb{Q}(\vec{x}) = S_n^2 + S_s^2 - \mathcal{w}^2$$

where S_n is the local stretching rate:

$$S_n(\vec{x}) = \frac{\partial \bar{u}}{\partial x} - \frac{\partial \bar{v}}{\partial y}$$

S_s is the local shearing rate:

$$S_s(\vec{x}) = \frac{\partial \bar{v}}{\partial x} + \frac{\partial \bar{u}}{\partial y}$$

and $\mathcal{w}$ is the local vorticity:

$$\mathcal{w}(\vec{x}) = \frac{\partial \bar{v}}{\partial x} - \frac{\partial \bar{u}}{\partial y}$$

$\mathbb{Q}$ is defined in such a way that positive values mean that the local flow is dominated by deformation, while negative values reflect a flow dominated by local rotating elements. Figure S4 shows the $\mathbb{Q}$ values obtained for the 4W configuration and using both turbulent models. We can see in the corresponding 2D contour plots that both models yield a similar flow field: air enters through the rear windows (denoted with white arrows) forming vortices inside the bus (denoted with asterisks) and leaves the bus through the front windows forming vortices outside the bus: this is the pumping back-to-front effect commented in the main text. On the other hand, we can also observe some quantitative differences between the models; particularly, the $\kappa - \epsilon$ underpredicts the vorticity intensity in some parts inside the bus.

In the main text we also mentioned that we chose the air traveling time, $t = L/U$, as the scaling parameter for the experimental 1:10 model. However, this scaling option will reduce the Reynolds number by a factor of 100 even though the flow is still turbulent. To see what happens if we change Re by a factor of 100, we computed the 2D flow field for the 4W configuration at $Re = 2 \times 10^4$ and 2×10^6. The results are plotted in Figure S5 where the blue lines denote the average streamlines, and the color map indicates the value of the turbulent kinetic viscosity μ_T [m^2/s]. Interestingly, notice that the average flow structure is similar in both cases as well as the spatial distribution of μ_T; however, the magnitude of μ_T changes by a factor of 100.

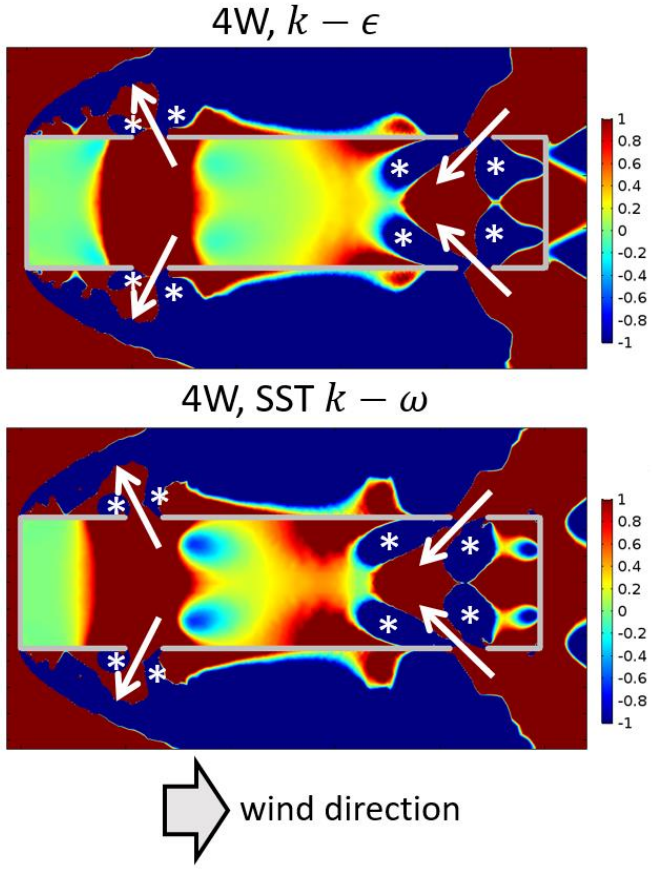

Figure S4. 2D Okubo-Weiss parameter $\mathbb{Q}$ computed for the 4W configuration and using both turbulent models. The wind direction (50km/h) is indicated with an arrow. Bus walls are marked with gray lines.

S5. General map of the mean age of air

In Figure 3 of the main text we included the 3D-contours of the mean age of air, $\mathcal{A}$, for the 4W and FW cases, specifically for the in-cabin zone. Here we present a general view of the mean age of air appearing in the whole computational domain. In Figure S6 we can see that $\mathcal{A}=0$ at the inlet wall, which is where air enters the domain, while at the outlet wall it has a value of 5.67s. This value agrees with the time it takes the air to travel the whole computational domain and which is equal to 79.3[m]/13.9[m/s]=5.7s. Also note that the $\mathcal{A}$-contour surfaces form a tail at the back of the bus; this trailing wake happens because air is trapped inside the bus and in the vortices located at the rear of the bus (there is also a delay at the ground level due to the no-slip condition).

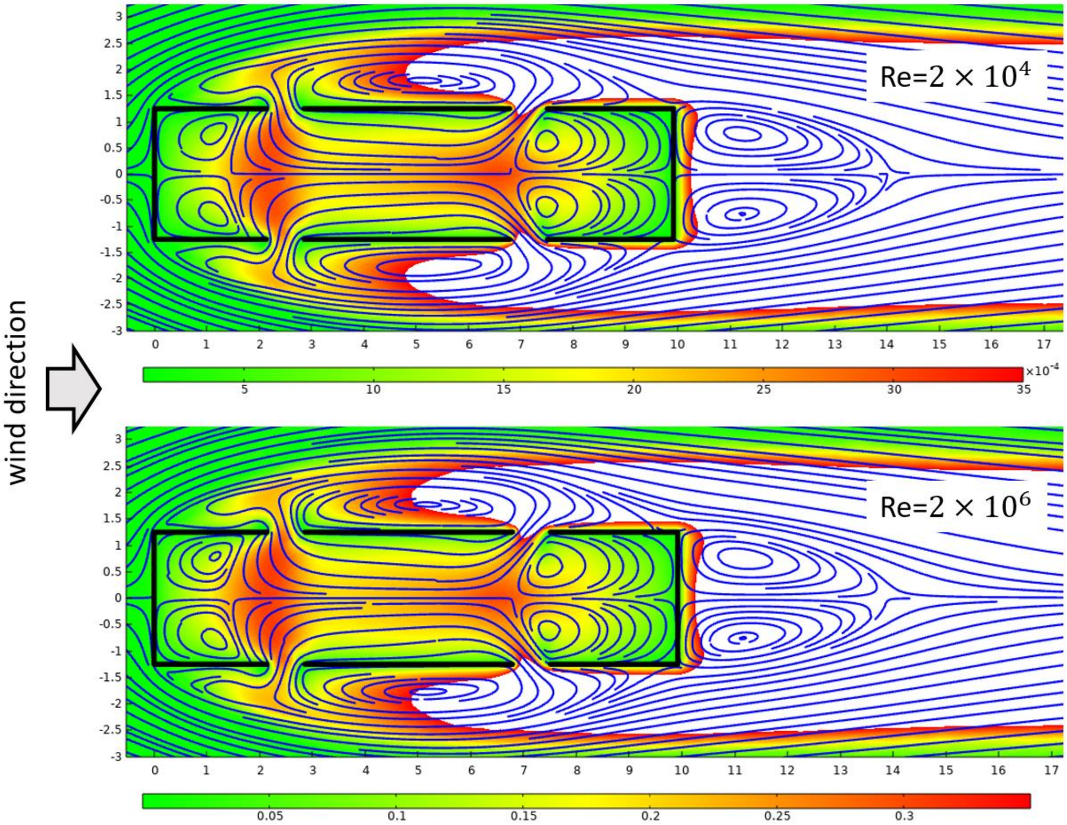

Figure S5. Average streamlines and turbulent kinematic viscosity [m^2/s] for two Reynolds numbers. The solid black lines denote the bus walls and correspond to the 4W configuration.

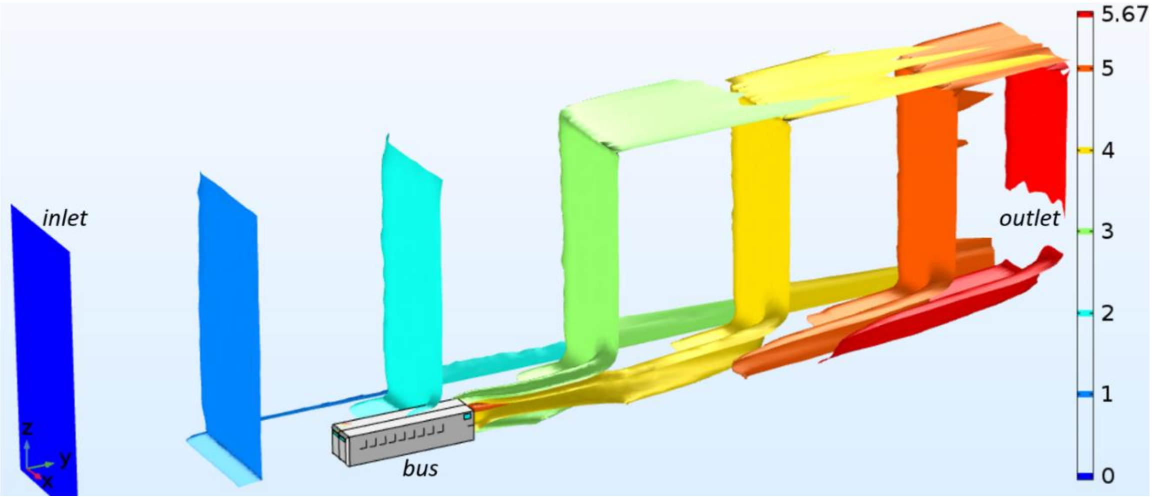

Figure S6. General view of the mean age of air obtained in the whole 3D computational domain.

S6. Driver's window in windward position

Figure S7 shows the 2D pressure map, together with some streamlines and the velocity field, found in a configuration where we included a driver's window in windward position (marked with asterisks). The back-to-front pumping effect at the lateral walls is still observed together with the negative pressure distribution inside the bus.

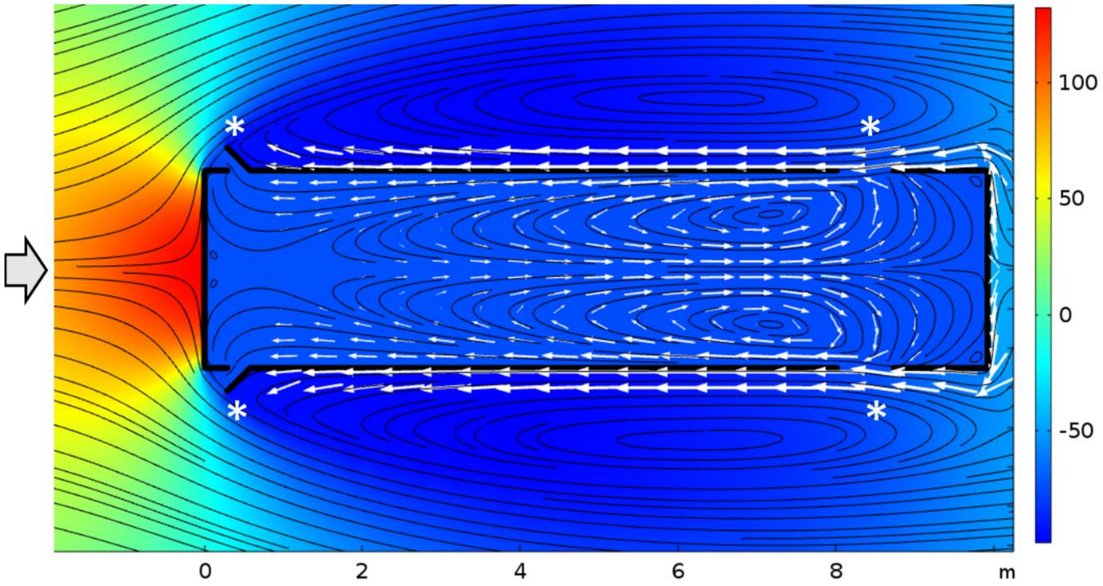

Figure S7. Fluid flow field and pressure [Pa] found in a 2D construction considering two driver's windows and two open windows at the rear (marked with asterisks). The incoming air is marked with an arrow. The bus walls are denoted with black lines.

S7. Comparison of the experimental expulsion rates in an empty and occupied bus

Figure S8 shows a comparison of the CO_2 amount measured inside the bus with and without passengers (manikins) for the 4W and FW cases. We can observe that the initial expulsion rate is larger (slopes of the curves) when the passengers are present, in accordance with the reduced age of air values obtained in the simulations including passengers.

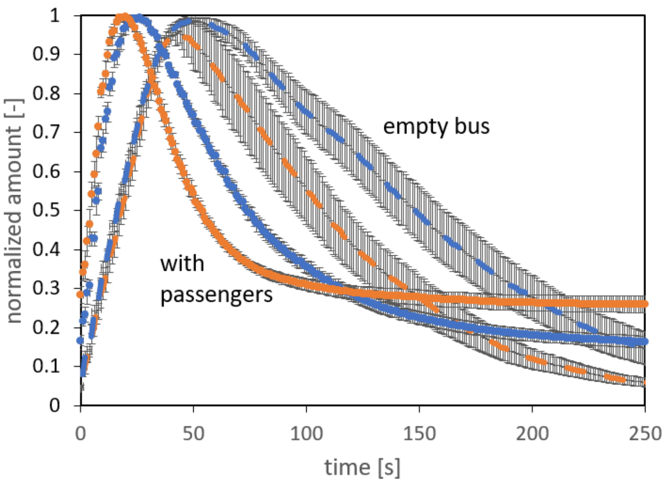

Figure S8. In-cabin normalized CO_2 concentration as a function of time detected at the middle part of the scale bus model with and without manikins. Orange curves correspond to the frontal FW case, while the blue curves denote the 4W lateral case.

S8. Age of air in an occupied bus: high-resolution manikins

In the main text we show that when the bus contains seated passengers, the mean age of air reduces from 50 to 32s in the frontal window configuration, that is, aerosols are expelled more rapidly from the bus when passengers are present. We ran an additional simulation using high-resolution manikins, but unfortunately, we didn't get a converged solution (error below 10^{-3}) after several days of computation (total elements were around 3.5×10^{6}). For the unique purpose of sharing these results having an associated error of 8%, we included here the corresponding age of air map; see Figure S9. Although the plot cannot be taken as a definite and rigorous result, the distribution looks similar to the plot included in the main text using low-resolution manikins, giving for this case an average age of air value of 44s and an average internal flow of 0.87m/s (for the empty bus the values were 50s and 0.8m/s, respectively).

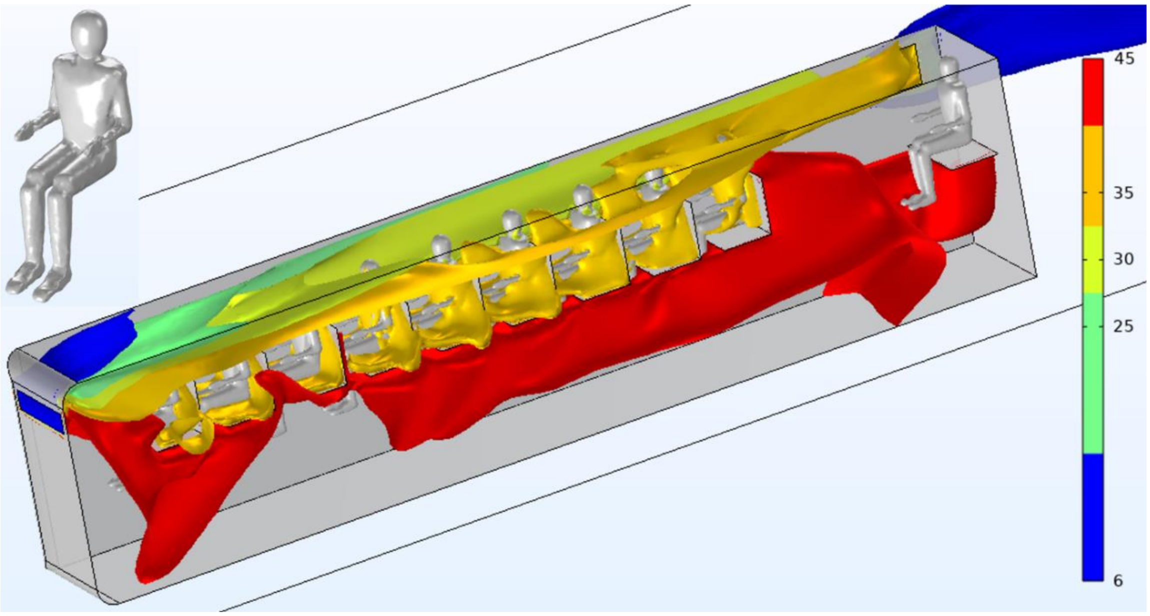

Figure S9. Age or air values [seconds] obtained for the FW configuration having high-resolution manikins. The detail of the manikin is shown in the inset.

Video legend: In the video “Windows movies.wmv” we show the dispersion of an aerosol “cloud” emitted inside the bus having different open windows configurations: 2W (2 open windows), 4W (4 open windows), AW (all windows open) and FW (one pair of frontal windows open together with one pair of lateral windows open at the back). The walls of the bus are denoted with white lines. Simulations were run in 2D using the SST k-ω and the concentration-based model. Color bar indicates the concentration level. IMPORTANT OBSERVATION: in the FW configuration aerosols are expelled completely in the shortest possible time.

References

[1] F.R. Menter and M. Kuntz, Adaptation of Eddy-Viscosity Turbulence Models to Unsteady Separated Flow Behind Vehicles, in: ``The Aerodynamics of Heavy Vehicles: Trucks, Buses, and Trains'', Lecture Notes in Applied and Computational Mechanics, McCallen R., Browand F., Ross J. (eds), Springer-Verlag 2004.

[2] H. Singh, D. F. Fletcher and J. J. Nijdam, An assessment of different turbulence models for predicting flow in a baffled tank stirred with a Rushton turbine. Chem. Eng. Sci 66, 5976 (2011).

[3] F. R. Menter, M. Kuntz and R. Langtry, Ten Years of Industrial Experience with the SST Turbulence Model, in: “Turbulence, Heat and Mass Transfer 4”, K. Hanjalic, Y. Nagano and M. Tummers (eds), Begell House, Inc. 2003.

[4] Yilin Deng, Jian Feng, Fulai Wan, Xi Shen and Bin Xu, Evaluation of the Turbulence Model Influence on the Numerical Simulation of Cavitating Flow with Emphasis on Temperature Effect. Processes 8, 997 (2020).

[5] Tian Li, Deng Qin and Jiye Zhang, Effect of RANS Turbulence Model on Aerodynamic Behavior of Trains in Crosswind. Chin. J. Mech. Eng. 32, 85 (2019).

[6] T. van Hooff, B. Blocken and G. J. F. van Heijst, On the suitability of steady RANS CFD for forced mixing ventilation at transitional slot Reynolds numbers. Indoor air 23, 236 (2012).

[7] Ahmadreza Haghnegahdar, Yu Feng, Xiaole Chen and Jiang Lin, Computational Analysis of Deposition and Translocation of Inhaled Nicotine and Acrolein in the Human Body with E-cigarette Puffing Topographies. Aerosol. Sci. Technol. 52, 483 (2018).

[8] F. Li, J. Liu, J. Ren, X. Cao and Y. Zhu, Numerical investigation of airborne contaminant transport under different vortex structures in the aircraft cabin. Int. J. Heat Mass Transfer 96, 287 (2016).
